# Estimation of Pyruvic acid in serum and saliva among healthy and potentially malignant disorder subjects – a stepping stone for cancer screening?

**DOI:** 10.4317/jced.52266

**Published:** 2015-10-01

**Authors:** Anithraj Bhat, Manohara Bhat, Kakarla Prasad, Dhiraj Trivedi, Swathi Acharya

**Affiliations:** 1 MBBS, Post Graduate, Department of Radiology, S.D.M. College of Medical Sciences & Hospital, Sattur, Dharwad-580009, India; 2 MDS, Asst. Professor, Department of Public Health Dentistry, KVG Dental College and Hospital, Sullia, D.K; 3 Professor & Head, Department of Public Health Dentistry, S.D.M. College of Dental Sciences & Hospital, Sattur, Dharwad-580009, India; 4 Professor & Head, Department of Biochemistry, S.D.M. College of Dental Sciences & Hospital, Sattur, Dharwad-580009, India; 5 Post Graduate, Department of Pharmacology, Vijayanagara Institute of Medical Sciences, Bellary, India

## Abstract

**Background:**

According to Warburg’s effect, the rate of glycolysis increases in cancerous cells. This will increase overall levels of pyruvic acid. The present on-going study was conducted to estimate the levels of pyruvic acid in saliva and serum in normal, oral PMD subjects.

**Material and Methods:**

A total of 50 subjects in healthy, PMD of the oral cavity individuals were selected based on clinical and histological criteria. Collected saliva and serum samples were subjected to pyruvic acid level estimation using biochemical analysis.

**Results:**

Of the 50 participants 25 (13: Males; 12: Females) & 25 (16: Males; 9: Females) were PMD group. Independent samples t test showed statistically significant difference in serum & salivary pyruvic acid level in between 2 groups (*p* < 0.001 respectively).

**Conclusions:**

Estimation of pyruvic acid showed sequential increase in the level in PMD group compared to healthy. Hence the study results open new direction in cancer screening.

** Key words:**Pyruvic acid, glycolysis, warburg’s effect.

## Introduction

It has been well established by researchers that virtually all oral cancer are preceded by visible clinical changes in the oral mucosa, usually in the form of white or red patch. World Health Organization (WHO) (2005) changed the terminology of premalignant condition and lesion into poetically malignant disorder (PMD) (1). PMD includes many oral conditions like Oral leukoplakia, Oral lichen planus, Actiniccheilitis, Oral Submucous fibrosis and many more, out of which leukoplakia and erythroplakia are the most common ones. These diagnoses are still based on the white and red lesion in the oral cavity (1). Approximately 70% of oral carcinomas detected by visual inspection (2). At present there are many cancer screening techniques including routine clinical examination, toluidine blue staining, tissue auto fluorescence, oral brush biopsy (3). But these methods have their own limitations such as Acceptability, Affordability and Accessibility and also patients seeking attention, and delays in medical and dental practitioners referring patients for diagnosis and treatment have been noted as important factors for possible delay in diagnosis (4).

In spite of tremendous progress in the field of molecular biology there is yet no single marker that reliably enables to predict malignant transformation in an individual patient (1) in more objectively. Therefore there is a need to think a technique of screening which mask all the limitations of the present screening method. Among the products of metabolism, pyruvic acid is of great importance because it is an intermediary in carbohydrate, as well as in protein and in fat metabolism. None of the studies estimated the pyruvate changes in PMD and oral cancer. It has been observed that cancer cells frequently disclose increase glycolysis and depend largely on this metabolic pathway for generation of ATP to meet their energy requirements (5). However, whether the increase of glycolytic activity in cancer cells is mainly due to inherent metabolic alterations or due to anaerobic environment in the tumour tissues remains controversial (6,7).

Estimating the level of pyruvic acid in saliva and serum might reflect the spectrum of oral cancer. Hence the aim of the present on-going study is to estimate and compare the levels of pyruvic acid in saliva and serum in normal, oral PMD subjects.

## Material and Methods

Following approval from the SDM College of Dental Sciences and Hospital Ethics Committee, recruitment was via patients attending the oral medicine outpatient clinic at SDM College of Dental Sciences and Hospital. Data were collected over 2 month period, by one student researchers and one academic researcher. A convenience sample was used, with as many patients recruited as possible within the available timeframe.

Two groups, i.e. Healthy & PMD were selected based on confirmed clinical and histological reports. Subjects above 40 years of age and those who give informed consent were selected for the study. Those who give negative clinical report were recruited in the Healthy group, whereas subjects with positive clinical and histological reports, irrespective of grades and stages were recruited in the PMD group. Oral Sub Mucous Fibrosis was identified only through clinical diagnostic method, as biopsy was contraindicated in those cases. Newly diagnosed PMD were included in the study. Exclusion criteria included systemic diseases like Cardiac diseases, diabetes and other carbohydrate metabolic disorders and subjects under chemotherapy, radiotherapy and surgery. Demographic data and Data regarding the personal habits, medication, past dental and medical history were collected from all the two groups. Once the subject selected for the study, subjects were asked to give 3ml of unstimulated saliva and 5 ml of venous blood.

Immediately saliva and blood samples were kept in deep freeze to reduce the rate of biochemical and bacterial reactions and transferred to the biochemical laboratory within one hour of sample collection.

Biochemical Procedure.

Blood Pyruvate Quantification is performed by the modified Di Nitro Phenyl Hydrazine (DNPH) procedure proposed by Landon J, Fawcett JK, and Wynn V. whereas in Salivary Pyruvate Estimation first 2 steps were bypassed due to fewer amounts of protein levels in saliva when compared to serum.

On comparing Healthy with PMD, Independent sample t test was used to measure the statistical significant difference between two groups.

## Results

In total,50 subjects were recruited. Of the participants 25 (13: Males; 12: Females) & 25 (16: Males; 9: Females) were PMD group. The mean age of healthy group was 53.8 and PMD group was 52.6 ( Table 1).

**Table 1:**
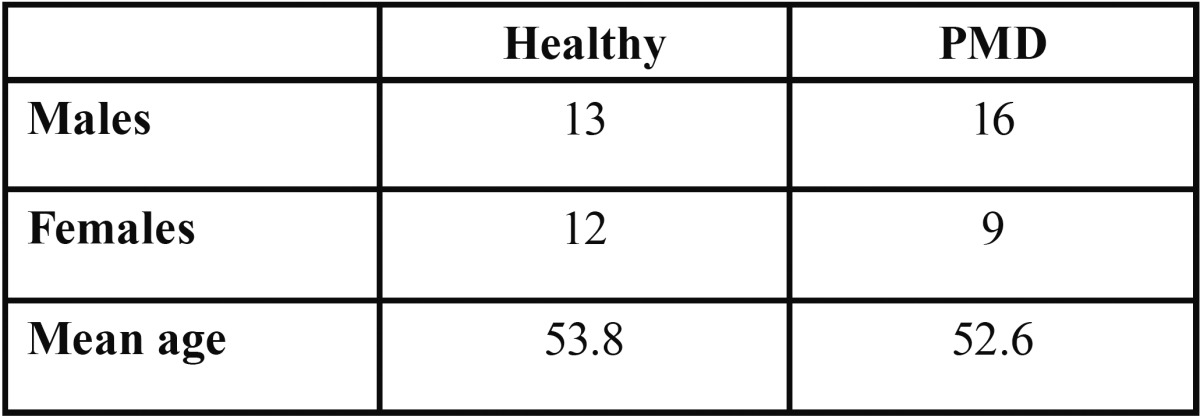
Gender and mean age distribution.

-Comparison of Serum Pyruvic acid levels of Healthy with PMD

**Figure 1:**
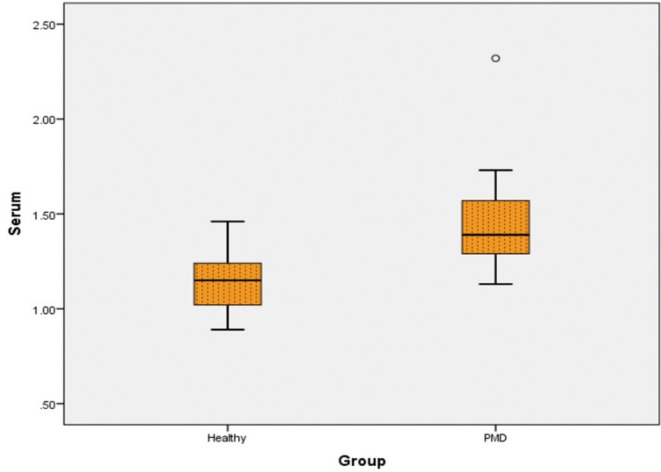
Pyruvic acid levels in serum of Healthy and PMC individuals.

Serum pyruvic acid levels of healthy group was 1.15 ± 0.15 and for PMD, it was 1.45 ± 0.25. Independent Sample t test showed statistically significant diff. between the groups (*P* < 0.001) ( Table 2) (Fig. 1).

**Table 2:**
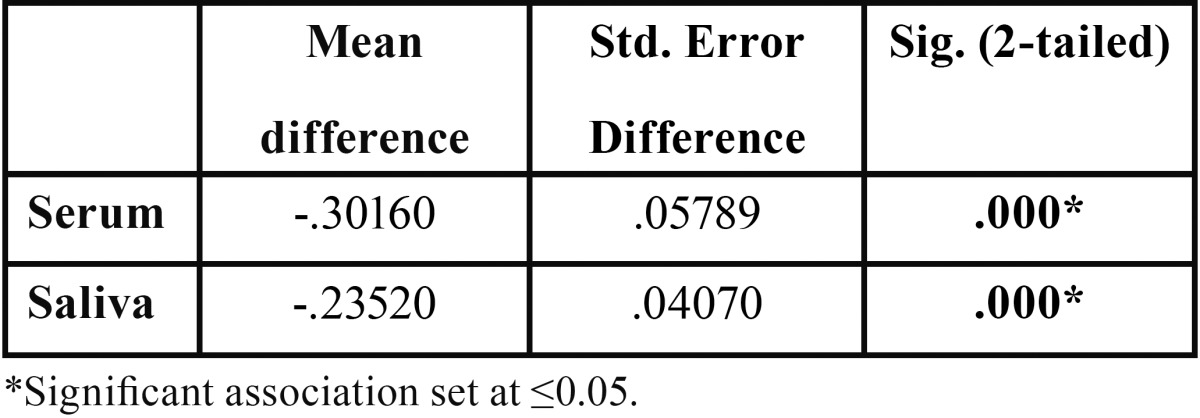
Independent Samples Test on Healthy and PMD group.

-Comparison of Salivary Pyruvic acid levels of Healthy with PMD

**Figure 2:**
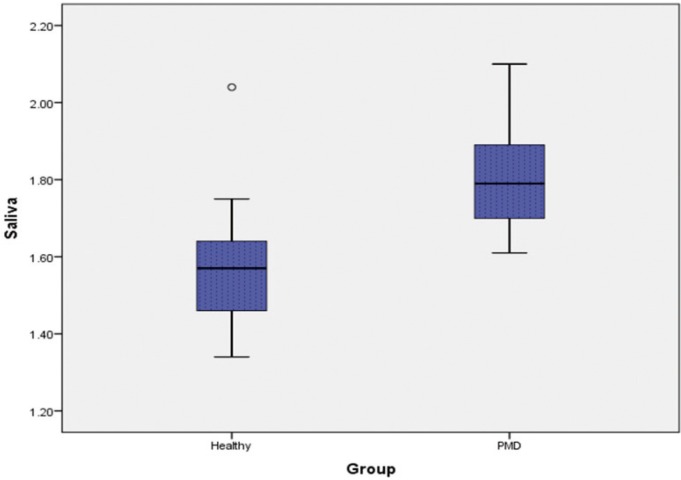
Pyruvic acid levels in saliva of Healthy and PMC individuals.

A clear differentiation between the Healthy and PMD was seen (with *P* < 0.001 for saliva) with the much greater mean Pyruvic acid levels in PMD subjects (Healthy: (1.57 ± 0.15) & PMD: (1.8 ± 1.4)) ( Table 2) (Fig. 2).

## Discussion

In normal physiologic condition, Pyruvic acid produced by Glycolysis cycle will be utilized by Kreb’s Cycle in mitochondria for further ATP production. ATP is synthesized by oxidative phosphorylation in mitochondria; which is efficient metabolic process and it produces more ATP molecules from a given amount of glucose when compared to glycolysis. However, this method of production of ATPs is compromised in cancer cells. Hence cells are able to adapt alternative metabolic pathway such as increased glycolytic pathway to maintain their energy supply. Cancer cell shows changes not only in the glycolytic pathway but also the Krebs cycle, b-oxidation and anabolic metabolism in general are reoriented to respond to the new primary function of the cell (i.e. Uncontrolled proliferation) by providing not only energy, but also the synthesis of nucleotides and amino and fatty acids (8-12).

Mitochondrial respiratory function can be compromised by many factors. Some of the factors include mutations in mitochondrial DNA (mtDNA), malfunction of the electron transport chain, aberrant expression of enzymes involved in energy metabolism, and insufficient oxygen available in the cellular microenvironment. Mitochondrial DNA contains displacement loops, which has the coding sequence, which is responsible the production of 13 important protein components of mitochondrial respiratory complexes (6).

Warburg’s effect can also be initiated by Hypoxia as the rate of angiogenesis is comparatively less than that of the rate of growth of tumour, this will induce depleted oxygen environment in neoplastic area and induces alternative respiratory method for the energy production and its survival (6) i.e. Glycolysis pathway. Hence Glycolysis is a central metabolic pathway that finely regulates cell proliferation by adapting the cancer cell’s metabolism to the conditions of its current selective situation (13-17).

Increased glycolytic rate produces more of its end products like Pyruvic acid and Lactic acid. As lactic acid is unstable compound, it converts back to pyruvate. This leads to increase the overall the levels of pyruvic acid. This excess pyruvic acid either leaches into blood or local region (oral cavity). Therefore quantification of pyruvic acid might give the proportional level of severity of the PMD. Hence we used Quantification of Pyruvic acid for this study.

The blood pyruvic acid estimation was performed by the hydrazin method of Lu and of Friedemann-Haugen. The mean blood pyruvate level in the present study came up to 1.15 ± 0.15 mg which is similar to the level estimated using a hydrazone method by Bueding and Wortis i.e. 0.98 ±0.09 mg in 60 fasted subjects (10).

Salivary pyruvic acid level for normal individuals was not established till now but in the present study the level came up to 1.460 ± 0.64. This is slightly elevated than the serum levels, as oral bacteria belongs to prokaryotic type of family, they do not possess mitochondria (18) and uses only glycolysis pathway for their energy requirement and survival.

The present study result showed elevated Pyruvic acid level in saliva than in serum, this may be because in serum pyruvic acid levels get balanced with the process of Cori’s cycle occurring in liver and another reason might be leaching out of Pyruvic acid may be more common to local region (Saliva) than the systemic (Serum). Further studies require in this direction to prove this hypothesis. The present research is an on-going study,we did not consider the severity of PMD as there was no common Stratification system for all type of PMD. This may have some influence on the study result.

Future directions 

• Warburg’s effect is common in most of the cancers; this principle can be used not only in screening procedures of the oral cancer but also other cancers.

• Saliva will play a major role in cancer detection as it provides noninvasive significant results. Salivary estimation of Pyruvic acid is comparatively more economical and less technique sensitive than the Serum estimation.

## Conclusions

The present study is an ongoing research with limited number of sample; continuation of the full-fledged present study with larger samples is going on in our departments. There were significant increases in pyruvic acid levels in the PMD, which can be further utilized for the development of a screening tool for the PMD or malignancy. The spectrum of cancer begins with healthy (Normal) tissue to PMD at subclinical state to full blown malignancy. This progression is shown in an ordinal fashion. Thus further studies should be performed to set the threshold of pyruvic acid level in each group at different stages.
